# Triglyceride glucose index and mortality in tracheally intubated patients: a MIMIC-IV retrospective cohort study

**DOI:** 10.1371/journal.pone.0324162

**Published:** 2025-05-21

**Authors:** Weide Lin, Junfan Chen, Feitao Cai

**Affiliations:** 1 Department of Anesthesiology, The First Hospital of Putian City, Putian, China; 2 Department of Medical Equipment Department, The First Hospital of Putian City, Putian, China; 3 Department of Ultrasonography, The First Hospital of Putian City, Putian, China; University of Diyala College of Medicine, UNITED STATES OF AMERICA

## Abstract

Tracheal intubation is crucial in ICU treatment but poses risks of complications and mortality. Simple and effective indicators for assessing mortality risk in intubated ICU patients are needed. This study analyzed 5,915 intubated ICU patients from the Medical Information Mart for Intensive Care 3.0 database. Multivariable Cox regression and two-stage linear regression models were used to assess the relationship between triglyceride glucose (TyG) index levels and ICU and in-hospital mortality rates. High TyG levels significantly correlated with increased mortality risk (HR range 1.34–1.44, p < 0.01). The association was linear with ICU mortality but nonlinear with in-hospital mortality. TyG levels ≥9.2 significantly increased in-hospital mortality risk (HR: 1.277, 95% CI: 1.114–1.464, p < 0.001). Higher TyG indices were associated with a higher risk of ICU and in-hospital mortality, emphasizing the importance of these findings for early prevention or timely intervention in intubated ICU patients.

## 1. Introduction

Tracheal intubation is a key technique in critical care medicine, typically used for patients who require mechanical ventilation support. It is considered the gold standard procedure for airway management [1]. Many tracheal intubations are performed in ICUs worldwide each year [2]. These patients often have severe multi-organ dysfunction, making timely tracheal intubation crucial for improving their prognosis. Clinically, tracheal intubation is not only used for airway protection and clearing respiratory secretions but also aids in the diagnosis and treatment of various diseases. However, despite its vital role in medical practice, tracheal intubation carries potential risks and complications, including tracheal injury and stenosis [3]. Tracheal intubation is a significant physiological and psychological stress event for patients, and studies have shown that this airway management measure is one of the main procedures leading to mortality and severe complications in the ICU [4]. Therefore, tracheal intubation is a common life support method in the ICU and is crucial for the treatment of critically ill patients [5]; however, it also has a higher risk of mortality and complications, reflecting the complexity of clinical management. Consequently, finding easily accessible and low-tech predictive indicators of in-hospital mortality in patients undergoing tracheal intubation in the ICU is particularly important.

Insulin resistance (IR) refers to the decreased efficiency of insulin in promoting glucose uptake and utilization [6]. It is closely associated with pathological states such as systemic inflammation, endothelial dysfunction, increased oxidative stress, and heightened prothrombotic tendencies [7,8]. The gold standards for assessing IR are the euglycemic hyperinsulinemic clamp test and intravenous glucose tolerance test; however, their invasive nature and high cost limit their widespread use in clinical practice [9]. Homeostasis Model Assessment for Insulin Resistance (HOMA-IR), a commonly used tool for assessing IR, is not applicable to patients undergoing insulin therapy or those with β-cell dysfunction [9,10]. In contrast, the triglyceride-glucose (TyG) index overcomes this limitation [11,12] and can be assessed solely based on a patient’s fasting blood glucose and fasting triglyceride levels [11,13]. The TyG index has been confirmed as a simple alternative indicator of insulin resistance with the advantages of cost-effectiveness and reliability [14]. It demonstrates superior diagnostic accuracy for insulin resistance in both patients with and without diabetes and is applicable across all populations, including those receiving insulin therapy [11].

During tracheal intubation, the release of a large amount of inflammatory mediators and oxidative stress imbalance are key factors leading to insulin resistance. Therefore, further exploration of the relationship between insulin resistance and clinical outcomes in intubated patients is crucial. This study aimed to investigate the impact of the TyG index on ICU and in-hospital mortality in intubated ICU patients, potentially aiding in the identification of higher-risk individuals who may benefit from closer monitoring or early intervention.

## 2. Materials and methods

### 2.1. Study population

This was a retrospective cohort study utilizing the Medical Information Mart for Intensive Care-IV (MIMIC-IV) database, which contains comprehensive medical records of tens of thousands of patients admitted to the ICU at the Beth Israel Deaconess Medical Center between 2008 and 2022. Its creation was approved by the Institutional Review Boards of both Beth Israel Deaconess Medical Center and Massachusetts Institute of Technology Affiliates. We selected data from 5,915 intubated patients from this database for analysis. The first author, Weide Lin, gained access to the database after completing the online review and agreeing to a data use agreement (Certification Number: 62407435). The data were accessed for research purposes between October 3, 2024, and November 6, 2024. This study adhered to the ethical guidelines of the Declaration of Helsinki. Ethical approval was waived by the Institutional Review Board (IRB) because the study utilized de-identified data from the publicly available MIMIC-IV database, which does not involve direct contact with human participants. Informed consent was not required because of the de-identified nature of all patient data. The authors did not have access to any information that could identify individual participants during or after data collection, as all personally identifiable information had been removed from the dataset. Furthermore, the reporting of this study strictly followed the Strengthening the Reporting of Observational Studies in Epidemiology (STROBE) reporting guidelines [15].

### 2.2. Covariates and outcome

We used a Structured Query Language (SQL) for data extraction. The covariates extracted from the MIMIC-IV database included sex, age, race, body mass index (BMI), heart rate, systolic blood pressure (SBP), diastolic blood pressure (DBP), respiratory rate (Resp), oxygen saturation (SpO_2_), Hamoglobin (Hb), white blood cell (WBC), neutrophilic granulocyte (NE), prothrombin time (PT), international normalized ratio (INR), activated partial thromboplastin time (APTT), myocardial infarction (MI), cerebrovascular disease (CVD), peripheral vascular disease (PVD), chronic obstructive pulmonary disease (COPD), peptic ulcer disease (PUD), diabetes mellitus (DM), liver disease, renal disease, mechanical ventilation (MV), Acute Physiology Score III (APS III), Oxford Acute Severity of Illness Score (OASIS). TyG was calculated using the following formula: TyG index = Ln (fasting triglycerides [mg/dL] × fasting glucose [mg/dL])/2 [16]. Additionally, we divided the TyG index into four quartiles, with ICU and in-hospital mortality as study outcomes.

### 2.3. Statistical analysis

Continuous variables with a normal distribution are depicted as the mean ± standard deviation (SD) and were compared using Student’s t-test or one-way ANOVA. For continuous variables that did not follow a normal distribution, data were presented as median and interquartile range (IQR), and statistical analysis was performed using the Kruskal–Wallis H test. Categorical variables were expressed as proportions (%) and were tested using the chi-square test or Fisher’s exact test. Additionally, there was significant insufficiency in some covariates: NE was missing in 27.57% of the cases, whereas BMI was missing in 17.06%. The remaining covariates had minimal missing data, with rates ranging from 0.10% to 4.19%. To address this issue, we employed multiple imputations for the missing covariates. Specifically, we generated five sets of imputed values for missing data. Subsequently, Cox regression and subgroup analyses were conducted using one of the imputed datasets.

We employed univariate linear regression and multivariate Cox regression analyses to assess the relationship between the TyG index, ICU and in-hospital mortality rates. In the multivariate model, we included variables of clinical interest as well as covariates with a p-value less than 0.1 in the univariate analysis. Additionally, we selected other potential confounding factors based on the criterion of a change-in-effect estimate > 10%. An extended Cox model was used for all models. Initially, we conducted unadjusted analyses, followed by Model 1, which was adjusted for age and sex; Model 2 further included BMI, heart rate, SBP, DBP, Resp, Spo_2,_ Hb, WBC, NE, PT, INR, APTT, MI, CVD, DM, liver disease, renal disease, MV, APS Ⅲ, and OASIS in addition to the variables in Model 1; and Model 3 included race, PVD, COPD, and PUD in addition to the variables in Model 2.

We first applied a generalized additive model to explore the association between the TyG index, ICU and in-hospital mortality rates [17,18]. If nonlinear associations were observed, a two-piecewise linear regression model was used to assess the threshold effect of the TyG index, which was completed using a smooth curve plot. When the relationship between the TyG index, ICU and in-hospital mortality rates was revealed through a smooth curve, we used a recursive method to automatically determine the threshold and analyzed it based on the maximum likelihood method [17]. Additionally, we performed a linear trend test for the median values of various TyG index categories as continuous variables in the model.

To determine whether the relationship between TyG index levels, ICU and in-hospital mortality is stable in the intubated population, we conducted interaction and subgroup analyses for age (<65 versus ≥65), sex (female versus male), BMI (<25 years, between 25–30 years, and >30 years), MI (yes versus no), COPD (yes versus no), and MV (yes versus no).

All analyses were performed using the statistical software package R (version 4.4.2, R Foundation for Statistical Computing, Vienna, Austria) and Free Statistics software version 2.0. P values < 0.05 (two-sided) were considered statistically significant [19].

## 3. Results

### 3.1. Participant selection

**Fig 1:**
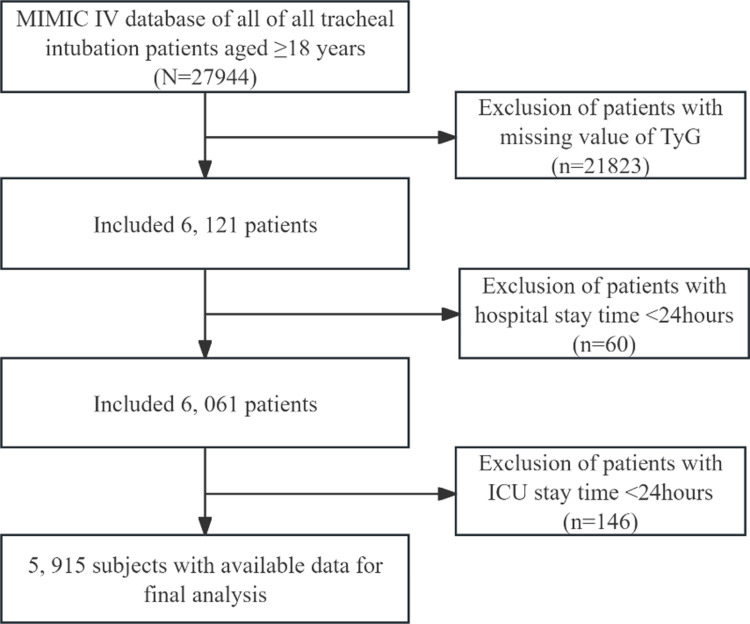
Schematic representation of the participant selection process and distribution of participant groups. This schematic illustrates the process of participant selection and the distribution across different participant groups. The flowchart detailed the inclusion and exclusion criteria, ultimately showing that 5, 915 subjects had available data for final analysis.

We identified 27,944 intubated patients using MIMIC-IV 3.0. After excluding patients with missing TyG index data (21,823), those with a hospital stay of < 24 hours (60), and those with an ICU stay of < 24 hours (146), we finally included 5,915 participants in the analysis. Some patients had multiple ICU admissions; however, we only considered the patients who were admitted to the ICU for the first time. A flowchart of the inclusion and exclusion criteria is shown in Fig 1.

### 3.2. Baseline characteristics of the study population

Table 1 summarizes the basic demographic characteristics of the study participants stratified by the TyG index quartiles. The mean age of the cohort was 60.6 ± 15.6 years, with 64.7% being male and a majority being white. Compared with the other three groups, patients with a high TyG index were younger and had faster heart and respiratory rates, lower SpO_2_, higher WBC count, NE, APS III scores, OASIS scores, ICU mortality, and in-hospital mortality.

**Table 1:** Baseline characteristics of participants.

Variables	Total (n = 5915)	Q1 (n = 1478) (<8.83)	Q2 (n = 1467) (≥8.83, < 9.37)	Q3 (n = 1490) (≥9.37, < 10.03)	Q4 (n = 1480) (≥10.03)	*P* value
**Sex, n (%)**						0.107
Female	2090 (35.3)	513 (34.7)	557 (38)	516 (34.6)	504 (34.1)	
Male	3825 (64.7)	965 (65.3)	910 (62)	974 (65.4)	976 (65.9)	
**Age(years)**	60.6 ± 15.6	64.3 ± 15.9	61.9 ± 16.0	59.1 ± 14.4	57.3 ± 15.4	< 0.001
**Race/Ethnicity, n(%)**						< 0.001
White	3356 (56.7)	948 (64.1)	800 (54.5)	822 (55.2)	786 (53.1)	
Black	542 (9.2)	143 (9.7)	136 (9.3)	95 (6.4)	168 (11.4)	
Other	2017 (34.1)	387 (26.2)	531 (36.2)	573 (38.5)	526 (35.5)	
**BMI(kg/m**^**2**^)	30.2 ± 8.5	30.0 ± 8.1	30.2 ± 8.1	30.5 ± 9.2	30.4 ± 8.6	0.452
**Heart rate (bpm)**	72.7 ± 16.2	69.8 ± 15.9	72.4 ± 15.6	74.2 ± 16.8	74.5 ± 16.1	< 0.001
**SBP (mmHg)**	88.8 ± 16.8	90.0 ± 16.8	88.4 ± 16.3	89.2 ± 16.8	87.6 ± 17.4	< 0.001
**DBP (mmHg)**	47.0 ± 10.9	46.0 ± 10.1	46.6 ± 11.3	47.6 ± 10.8	47.8 ± 11.2	< 0.001
**Resp (bpm)**	13.2 ± 4.2	12.3 ± 3.6	12.8 ± 3.8	13.2 ± 4.4	14.5 ± 4.7	< 0.001
**Spo**_**2**_ **(%)**	91.2 ± 6.9	92.5 ± 5.3	91.9 ± 7.0	90.5 ± 7.4	90.0 ± 7.4	< 0.001
**Hb(g/dL)**	10.3 ± 2.4	10.2 ± 2.6	10.2 ± 2.5	10.3 ± 2.4	10.4 ± 2.3	0.073
**WBC(×10^9/L)**	10.0 (7.0, 13.7)	9.5 (7.1, 12.5)	9.7 (7.1, 13.7)	10.3 (7.3, 14.1)	10.7 (6.6, 14.3)	0.001
**NE(×10^9/L)**	9.3 (6.0, 13.9)	9.2 (6.3, 13.3)	9.5 (6.3, 15.2)	9.3 (6.4, 13.5)	9.6 (5.1, 13.9)	0.048
**PT (s)**	14.6 ± 5.0	14.7 ± 4.8	14.7 ± 5.0	14.4 ± 5.4	14.4 ± 4.6	0.344
**INR (s)**	1.3 ± 0.5	1.3 ± 0.5	1.3 ± 0.5	1.3 ± 0.5	1.3 ± 0.4	0.688
**APTT (s)**	31.4 ± 12.4	31.5 ± 13.0	32.0 ± 15.0	30.4 ± 8.9	31.8 ± 11.8	0.002
**MI, n (%)**						< 0.001
No	4649 (78.6)	1133 (76.7)	1108 (75.5)	1199 (80.5)	1209 (81.7)	
Yes	1266 (21.4)	345 (23.3)	359 (24.5)	291 (19.5)	271 (18.3)	
**CVD, n (%)**						< 0.001
No	4338 (73.3)	1005 (68)	1062 (72.4)	1122 (75.3)	1149 (77.6)	
Yes	1577 (26.7)	473 (32)	405 (27.6)	368 (24.7)	331 (22.4)	
**PVD, n (%)**						0.108
No	5259 (88.9)	1326 (89.7)	1280 (87.3)	1325 (88.9)	1328 (89.7)	
Yes	656 (11.1)	152 (10.3)	187 (12.7)	165 (11.1)	152 (10.3)	
**COPD, n (%)**						< 0.001
No	4496 (76.0)	1107 (74.9)	1150 (78.4)	1047 (70.3)	1192 (80.5)	
Yes	1419 (24.0)	371 (25.1)	317 (21.6)	443 (29.7)	288 (19.5)	
**PUD, n (%)**						< 0.001
No	5714 (96.6)	1404 (95)	1426 (97.2)	1442 (96.8)	1442 (97.4)	
Yes	201 (3.4)	74 (5)	41 (2.8)	48 (3.2)	38 (2.6)	
**DM, n (%)**						< 0.001
No	4099 (69.3)	1186 (80.2)	1097 (74.8)	971 (65.2)	845 (57.1)	
Yes	1816 (30.7)	292 (19.8)	370 (25.2)	519 (34.8)	635 (42.9)	
**Liver disease, n (%)**						0.002
No	4795 (81.1)	1237 (83.7)	1188 (81)	1213 (81.4)	1157 (78.2)	
Yes	1120 (18.9)	241 (16.3)	279 (19)	277 (18.6)	323 (21.8)	
**Renal disease, n (%)**						< 0.001
No	4824 (81.6)	1259 (85.2)	1207 (82.3)	1229 (82.5)	1129 (76.3)	
Yes	1091 (18.4)	219 (14.8)	260 (17.7)	261 (17.5)	351 (23.7)	
**MV, n (%)**						< 0.001
No	2833 (48.3)	748 (51.5)	722 (49.8)	727 (49)	636 (43.1)	
Yes	3032 (51.7)	705 (48.5)	729 (50.2)	757 (51)	841 (56.9)	
**APS**Ⅲ****	54.3 ± 24.9	48.3 ± 22.9	51.5 ± 23.1	56.9 ± 27.0	60.5 ± 24.6	< 0.001
**OASIS**	37.0 ± 8.2	35.7 ± 8.1	36.5 ± 8.4	37.6 ± 8.6	38.0 ± 7.5	< 0.001
**ICU Mortality, n(%)**						< 0.001
No	5203 (88.0)	1337 (90.5)	1293 (88.1)	1313 (88.1)	1260 (85.1)	
Yes	712 (12.0)	141 (9.5)	174 (11.9)	177 (11.9)	220 (14.9)	
**In-hospital Mortality, n(%)**						< 0.001
No	4951 (83.7)	1275 (86.3)	1218 (83)	1261 (84.6)	1197 (80.9)	
Yes	964 (16.3)	203 (13.7)	249 (17)	229 (15.4)	283 (19.1)	

**Notes:** Continuous variables are presented as mean ± SD or median (quartile), while categorical variables are presented as absolute numbers (percentages).

**Abbreviations:** BMI, body mass index; SBP, systolic blood pressure; DBP, diastolic blood pressure; Resp, respiratory; Spo_2,_ pulse oximetry derived oxygen saturation; Hb, hemoglobin; WBC, white blood cell; NE, neutrophil; PT, prothrombin time; INR, international normalized ratio; APTT, activated partial thromboplastin time; MI, myocardial infarction; CVD, cerebrovascular disease; PVD, peripheral vascular disease; COPD, chronic obstructive pulmonary disease; PUD, peptic ulcer disease; DM, diabetes mellitus; MV, mechanical ventilation; APSIII, acute physiology score III; OASIS, oxford acute severity of illness score; ICU mortality, intensive care unit mortality.

### 3.3. The correlation between TyG and all-cause mortality

Univariate analyses of ICU and in-hospital mortality rates in intubated patients are shown in S1 Table. Sex, age, BMI, heart rate, SBP, DBP, Resp, Spo_2_, Hb, WBC, NE, PT, INR, APTT, MI, DM, liver disease, renal disease, MV, APS III, OASIS, and TyG were risk factors for ICU mortality (P < 0.05), whereas sex, age, heart rate, SBP, DBP, Resp, Spo_2_, Hb, WBC, NE, PT, INR, APTT, MI, CVD, DM, liver disease, renal disease, MV, APS III, OASIS, and TyG were risk factors for in-hospital mortality (P < 0.05). The K-M curves showed a significant correlation between TyG and the risks of ICU and in-hospital mortality (S1 Fig).

**Fig 2:**
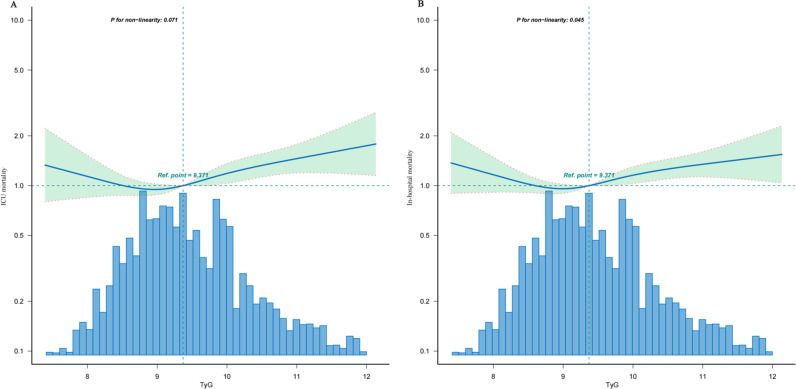
A Linear dose-response relationship between TyG and ICU mortality. The linear dose-response relationship between TyG and ICU mortality is presented. Adjustments were made for sex, age, race, BMI, heart rate, SBP, DBP, Resp, Spo_2,_ Hb, WBC, NE, PT, INR, APTT, MI, PVD, CVD, COPD, PUD, DM, Liver disease, Renal disease, MV, APSⅢ, OASIS. The blue line and green area represent the estimated values and their corresponding 95% confidence intervals, respectively. **B Nonlinear dose-response relationship between TyG and in-hospital mortality.** This figure shows the nonlinear relationship between TyG and in-hospital mortality, with adjustments for the same factors as in Fig 2A. The estimated values and 95% confidence intervals are depicted similarly, providing insights into how TyG influences in-hospital mortality risks.

The results of the multivariate Cox regression analysis indicate a significant positive correlation between TyG levels and the risk of ICU and in-hospital mortality. When TyG levels were treated as a continuous variable, there was a significant positive correlation between TyG and the risk of ICU mortality in both the unadjusted crude model (HR, 1.17 [95% CI 1.08–1.27], p < 0.001) and the fully adjusted Model 3 (HR, 1.16 [95% CI 1.06–1.26], p = 0.001). Similarly, TyG was significantly positively correlated with the risk of in-hospital mortality in both the unadjusted crude model (HR, 1.11 [95% CI 1.03–1.18], p = 0.003) and the fully adjusted Model 3 (HR, 1.11 [95% CI 1.03–1.2], p = 0.006). When TyG was treated as a categorical variable, in the unadjusted crude model, compared to TyG Q1, TyG Q4 had a higher risk of ICU mortality (HR, 1.53 [95% CI 1.24–1.89], p < 0.001); the results for TyG levels and in-hospital mortality risk were similar (HR, 1.38 [95% CI 1.15–1.65], p < 0.001). In the fully adjusted Model 3, compared to TyG Q1, TyG Q4 had a higher risk of ICU mortality (HR, 1.44 [95% CI 1.14–1.82], p = 0.002); the results for TyG levels and in-hospital mortality risk were similar (HR, 1.34 [95% CI 1.1–1.63], p = 0.004) (Table 2). Additionally, we found that in the Restricted Cubic Splines model, the association between TyG level and ICU mortality was linear (p = 0.071), whereas the association between TyG level and in-hospital mortality was nonlinear (p = 0.045) (Fig 2). In the threshold analysis, no significant association was observed between TyG levels and the risk of in-hospital mortality when TyG levels were less than 9.2 (HR, 0.831[95% CI 0.632–1.091], p = 0.1826); when TyG levels were 9.2 or higher, the risk of in-hospital mortality significantly increased (HR, 1.277 [95% CI 1.114–1.464], p < 0.001) (Table 3). This means that for each one-unit increment in TyG, the risk of in-hospital mortality rises by 27.7%.

**Table 2:** Relationships between the TyG index, ICU mortality, and in-hospital mortality across different models.

Variable	Crude model		Model Ⅰ		Model Ⅱ		Model Ⅲ	
	HR (95% CI)	*P-*value	HR (95% CI)	*P-*value	HR (95% CI)	*P-*value	HR (95% CI)	*P-*value
**ICU mortality**								
TyG as continuous	1.17 (1.08 ~ 1.26)	<0.001	1.24 (1.15 ~ 1.34)	<0.001	1.17 (1.07 ~ 1.27)	0.001	1.16 (1.06 ~ 1.26)	0.001
Quartiles								
Q1 (TyG** < **8.83)	**Ref**		**Ref**		**Ref**		**Ref**	
Q2 (8.83 ≤ TyG < 9.37)	1.21 (0.97 ~ 1.51)	0.092	1.24 (0.99 ~ 1.55)	0.058	1.34 (1.06 ~ 1.67)	0.012	1.23 (0.97 ~ 1.54)	0.082
Q3 (9.37 ≤ TyG < 10.03)	1.19 (0.96 ~ 1.49)	0.115	1.32 (1.05 ~ 1.65)	0.015	1.32 (1.05 ~ 1.67)	0.02	1.25 (0.99 ~ 1.58)	0.066
Q4 (TyG ≥ 10.03)	1.53 (1.24 ~ 1.89)	<0.001	1.74 (1.4 ~ 2.15)	<0.001	1.48 (1.18 ~ 1.87)	0.001	1.44 (1.14 ~ 1.82)	0.002
*P* for trend		<0.001		<0.001		0.002		0.003
**In-hospital mortality**								
TyG as continuous	1.11 (1.03 ~ 1.18)	0.003	1.18 (1.1 ~ 1.26)	<0.001	1.13 (1.05 ~ 1.21)	0.001	1.11 (1.03 ~ 1.2)	0.006
Quartiles								
Q1 (TyG** < **8.83)	**Ref**		**Ref**		**Ref**		**Ref**	
Q2 (8.83 ≤ TyG < 9.37)	1.18 (0.98 ~ 1.41)	0.088	1.22 (1.01 ~ 1.47)	0.035	1.24 (1.02 ~ 1.49)	0.028	1.16 (0.96 ~ 1.4)	0.135
Q3 (9.37 ≤ TyG < 10.03)	1.05 (0.87 ~ 1.27)	0.623	1.18 (0.97 ~ 1.42)	0.096	1.2 (0.98 ~ 1.46)	0.078	1.15 (0.94 ~ 1.41)	0.165
Q4 (TyG ≥ 10.03)	1.38 (1.15 ~ 1.65)	<0.001	1.6 (1.33 ~ 1.92)	<0.001	1.41 (1.16 ~ 1.72)	0.001	1.34 (1.1 ~ 1.63)	0.004
*P* for trend		0.003		<0.001		0.002		0.006

**Notes:** Crude model was not adjusted.

Model 1 was adjusted for age + sex.

Model 2 was adjusted for model 1 + BMI + heart rate + SBP + DBP + Resp + Spo_2_ + Hb + WBC + NE + PT + INR + APTT + MI + CVD + DM + liver disease + renal disease + MV + APSⅢ + OASIS.

Model 3 was adjusted for model 2 + race + PVD + COPD + PUD.

**Abbreviations:** BMI, body mass index; SBP, systolic blood pressure; DBP, diastolic blood pressure; Resp, respiratory; Spo_2,_ pulse oximetry derived oxygen saturation; Hb, hemoglobin; WBC, white blood cell; NE, neutrophil; PT, prothrombin time; INR, international normalized ratio; APTT, activated partial thromboplastin time; MI, myocardial infarction; CVD, cerebrovascular disease; DM, diabetes mellitus; MV, mechanical ventilation; APSIII, acute physiology score III; OASIS, oxford acute severity of illness score; PVD, peripheral vascular disease; COPD, chronic obstructive pulmonary disease; PUD, peptic ulcer disease.

**Table 3:** Threshold effect analysis of TyG levels on in-hospital mortality.

In-hospital mortality			
Turning point	HR	95%CI	*P*-value
TyG < 9.2	0.83	0.63,1.09	0.18
TyG ≥ 9.2	1.28	1.11,1.46	< 0.001
Likelihood Ratio test			0.02

HR, hazard ratio; CI, confidence interval. Adjustment factors included Sex, age, race, BMI, heart rate, SBP, DBP, Resp, Spo_2,_ Hb, WBC, NE, PT, INR, APTT, MI, PVD, CVD, COPD, PUD, DM, liver disease, renal disease, MV, APSⅢ, OASIS.

### 3.4. Subgroup analysis

**Fig 3:**
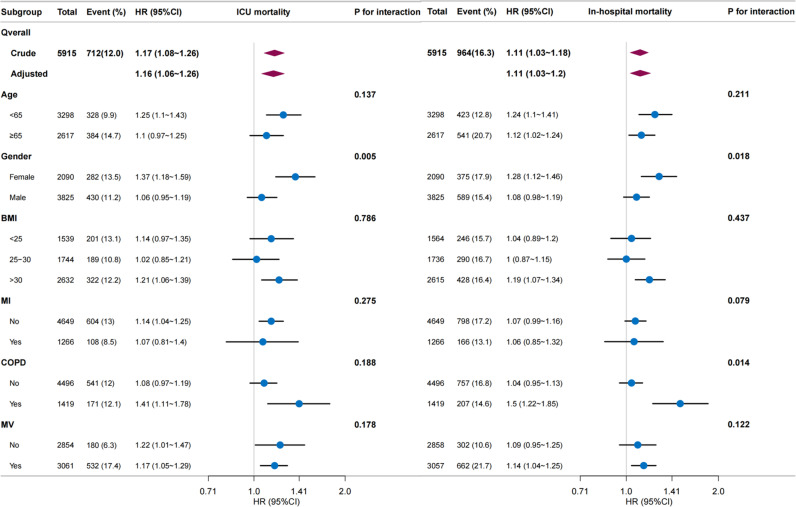
Subgroup analysis of the association between TyG and ICU mortality and in-hospital mortality. Subgroup analysis assessed the association between TyG and ICU mortality as well as in-hospital mortality. This analysis explored the impact of TyG on mortality across different subgroups, including factors such as age, gender, and comorbidities, to determine its consistency.

To determine whether a relationship between TyG levels, ICU and in-hospital mortality among intubated patients existed across different subgroups, we conducted stratified and interaction analyses based on age, sex, BMI, MI, COPD, and MV. The analysis results showed that there was an interaction effect of sex on both ICU and in-hospital mortality, and an interaction effect of COPD on in-hospital mortality (Fig 3).

### 3.5. Sensitive analysis

To validate the robustness of our findings, we conducted sensitivity analyses. The results showed that after excluding diabetic patients (n = 1816), the TyG index remained significantly associated with both ICU mortality and in-hospital mortality (S2 Table). Subsequently, we adjusted the model by including patients with hypertriglyceridemia (triglycerides ≥200 mg/dL) and those using lipid-lowering medications, and found that the results remained largely consistent (S3 Table).

## 4. Discussion

In this study, we observed that high TyG indices were associated with increased ICU and in-hospital mortality in intubated ICU patients. Further subgroup analysis revealed that the association between the TyG index, ICU and in-hospital mortality is more pronounced in female patients, while it does not show significant statistical significance in male patients. This sex difference may be related to various factors, including biological differences, hormone levels, and potential lifestyle and environmental factors, with specific reasons requiring further investigation. Notably, the TyG index exhibited a linear relationship with ICU mortality and a nonlinear relationship with in-hospital mortality. When the TyG index was below 9.2, there was no statistically significant association with the risk of in-hospital mortality among intubated ICU patients; however, when the TyG index exceeded 9.2, there was a positive correlation with the risk of in-hospital mortality.

Previous studies have indicated that the TyG index is associated with the prognosis of multiple systemic diseases [20] such as cardiovascular [21–24], cerebrovascular [25–27], and respiratory system diseases [28,29]. Additionally, the TyG index is associated with conditions such as depression [30,31], psoriasis [32], sepsis [33], and dementia [34]. Despite numerous studies on the TyG index, clinical evidence linking it to the outcomes in intubated patients remains limited and inconclusive.

IR leads to glucose metabolism disorders, which in turn can trigger hyperglycemia, potentially initiating inflammatory responses and increasing oxidative stress. Studies have shown that IR promotes an increase in advanced glycation end products and free radicals, leading to the inactivation of nitric oxide (NO). Abnormal secretion of NO associated with IR and excessive production of reactive oxygen species (ROS) due to IR are two reasons for impaired endothelial function [35,36]. Qing et al. [37] found in the tumor necrosis factor-α (TNF-α)-treated gulonolactone oxidase knockout mice model that vitamin C deficiency exacerbates IR, likely due to the anti-inflammatory effect of vitamin C, with its deficiency weakening this anti-inflammatory effect. Nieto-Vázquez et al. [38] reported that protein tyrosine phosphatase 1 B (PTP1B) could remove phosphate groups from the insulin receptor and related proteins. When the body is affected by the pro-inflammatory cytokine TNF-α, the protein expression of PTP1B increases, which may affect the normal function of insulin. In addition, the inflammatory factor interleukin (IL)-1 inhibits insulin signaling through phosphorylation of insulin receptor substrate and activates the mitogen-activated protein kinase pathway, both of which are mediated by its receptor IL-1R [39,40]. In summary, IR is a recognized marker of metabolic disorders and systemic inflammation [10]. The TyG index is widely recognized as a clinical surrogate marker for IR [41]. Previous studies have identified a close association between inflammation and the TyG index. Li et al. [42] found that high levels of inflammation and TyG index, either alone or in combination, increase the risk of colorectal cancer. Yan et al. [43] also reported that in nondiabetic American individuals aged < 60 years with normal weight, the TyG index was positively correlated with arthritis. Additionally, studies have found that a high TyG index is positively associated with an increased risk of periodontitis [44] and has been confirmed as an independent risk factor for severe pancreatitis, with elevated levels significantly associated with complications of severe and acute pancreatitis [45]. Previous observational studies have also shown that the TyG index positively correlates with inflammatory markers such as white blood cells and C-reactive protein [30].

A prospective cohort study of 100 consecutive patients reported that 57% of the mechanically ventilated patients who underwent tracheal intubation showed signs of acute laryngeal injury [46]. Studies have also indicated that prolonged endotracheal intubation may trigger a sustained inflammatory response [47], and both acute laryngeal injury and prolonged intubation are associated with inflammatory reactions. Patients undergoing tracheal intubation often experience hyperglycemia and acute glucose fluctuations [48]. Van Herpt et al. [49] noted that hyperglycemia and glucose variability are associated with mortality in mechanically ventilated ICU patients with COVID-19. The association between the TyG index and intubation may stem from their common involvement in inflammatory responses, hyperglycemia, or glucose fluctuations. Therefore, the relationship between the TyG index and intubation could be related to these factors.

Our study highlights the key role of the TyG index in assessing the prognosis of intubated patients. Personalized and timely risk assessments are crucial for devising precise treatment plans and improving patient outcomes. The TyG index, a prognostic biomarker easily obtainable from routine blood tests, facilitates rapid risk assessment.

Our study has certain limitations. First, as a retrospective study, it may be subject to selection bias and information bias; future research should employ prospective designs and account for these potential confounding factors to further validate our observations. Second, our study population was limited to intubated patients in the MIMIC-IV database, and whether these results are generalizable to all intubated patients requires further investigation. Third, although we rigorously adjusted for potential confounders, residual confounding may persist due to the inherent nature of retrospective studies, as not all relevant variables could be fully controlled. Despite these limitations, our study has several notable strengths. First, utilizing existing medical records enabled access to a comprehensive dataset, thereby enhancing the study’s statistical power. Second, the large sample size permitted simultaneous analysis of multiple variables, allowing for a thorough examination of relationships between various factors and outcomes. Third, as the data were derived from real-world clinical practice, the findings demonstrate enhanced applicability to actual clinical settings.

## 5. Conclusion

Our results indicate that higher TyG index levels are positively correlated with the risk of ICU and in-hospital mortality among intubated ICU patients. There was a nonlinear relationship between TyG index levels and in-hospital mortality among intubated ICU patients, and the risk significantly increased when TyG index levels exceeded 9.2.

## Supporting information

S1 Table Univariate analysis of in-hospital and ICU mortality rates among patients undergoing tracheal intubation. The analysis compares survivors and non-survivors in terms of in-hospital and ICU mortality. The table presents demographic data, vital signs, laboratory parameters, comorbidities, and severity scores.
(DOCX)

S2 Table Relationships between the TyG index, ICU mortality, and in-hospital mortality across different models. After excluding diabetic patients (n = 1816), the relationship between the TyG index and ICU mortality as well as in-hospital mortality.
(DOCX)

S3 Table Relationships between the TyG index, ICU mortality, and in-hospital mortality across different models. After including patients with hypertriglyceridemia (triglycerides ≥200 mg/dL) and those on lipid-lowering medications, the relationship between the TyG index and ICU mortality as well as in-hospital mortality.
(DOCX)

S1 Fig A Kaplan-Meier survival analysis of TyG and ICU mortality in patients with tracheal intubation. Kaplan-Meier survival analysis depicting the association between TyG and ICU mortality among patients who underwent tracheal intubation. The survival curves are stratified by TyG status, highlighting the differences in survival probabilities. **B Kaplan-Meier survival analysis of TyG and in-hospital mortality in patients with tracheal intubation.** This Kaplan-Meier survival plot examines the relationship between TyG and in-hospital mortality in patients with tracheal intubation. Indicating the impact of TyG and in-hospital mortality rates.
(TIF)
