# Supplementary material for: Triglyceride glucose index and mortality in tracheally intubated patients: a MIMIC-IV retrospective cohort study
Source: PLoS One. 2025 May 21;20(5):e0324162. doi: 10.1371/journal.pone.0324162 (PMC12094725; doi:10.1371/journal.pone.0324162)
Supplement: S1 Table — The analysis compares survivors and non-survivors in terms of in-hospital and ICU mortality. The table presents demographic data, vital signs, laboratory parameters, comorbidities, and severity scores. (DOCX) [file pone.0324162.s001.docx]

**TABLE S1 | Univariate analysis of in-hospital and ICU mortality rates among patients undergoing tracheal intubation.**

| **Variables** | **ICU mortality** | | **In-hospital mortality** | |
| --- | --- | --- | --- | --- |
|  | **HR(95%CI)** | ***P-*value** | **HR(95%CI)** | ***P-*value** |
| **Sex** | 0.84 (0.72,0.98) | 0.022 | 0.85 (0.75,0.97) | 0.016 |
| **Age** | 1.02 (1.01,1.02) | < 0.001 | 1.02 (1.02,1.03) | < 0.001 |
| **Race/Ethnicity** |  |  |  |  |
| White | Ref |  | Ref |  |
| Black | 1.09 (0.83,1.43) | 0.547 | 1.03 (0.81,1.31) | 0.812 |
| Other | 1.72 (1.48,2.01) | < 0.001 | 1.5 (1.32,1.72) | < 0.001 |
| **BMI** | 0.99 (0.98,1) | 0.011 | 0.997 (0.9892,1.0049) | 0.455 |
| **Heart rate** | 1.01 (1.0056,1.0144) | < 0.001 | 1.0079 (1.0041,1.0116) | < 0.001 |
| **SBP** | 0.98 (0.98,0.99) | < 0.001 | 0.99 (0.98,0.99) | < 0.001 |
| **DBP** | 0.98 (0.97,0.99) | < 0.001 | 0.98 (0.98,0.99) | < 0.001 |
| **Resp** | 1.08 (1.06,1.1) | < 0.001 | 1.06 (1.05,1.08) | < 0.001 |
| **Spo_2_** | 0.97 (0.97,0.98) | < 0.001 | 0.98 (0.97,0.98) | < 0.001 |
| **Hb** | 0.96 (0.94,0.99) | 0.02 | 0.93 (0.91,0.96) | < 0.001 |
| **WBC** | 1.01 (1.01,1.02) | < 0.001 | 1.01 (1.01,1.02) | < 0.001 |
| **NE** | 1.03 (1.02,1.04) | < 0.001 | 1.03 (1.02,1.04) | < 0.001 |
| **PT** | 1.05 (1.04,1.06) | < 0.001 | 1.04 (1.03,1.05) | < 0.001 |
| **INR** | 1.63 (1.48,1.78) | < 0.001 | 1.53 (1.4,1.66) | < 0.001 |
| **APTT** | 1.02 (1.01,1.02) | < 0.001 | 1.02 (1.01,1.02) | < 0.001 |
| **MI** | 0.65 (0.53,0.79) | < 0.001 | 0.74 (0.63,0.88) | < 0.001 |
| **PVD** | 1.07 (0.85,1.34) | 0.577 | 1.0096 (0.8244,1.2364) | 0.927 |
| **CVD** | 1.14 (0.97,1.34) | 0.12 | 1.19 (1.04,1.37) | 0.012 |
| **COPD** | 1.0091 (0.8495,1.1985) | 0.918 | 0.86 (0.74,1) | 0.058 |
| **PUD** | 1.08 (0.73,1.6) | 0.705 | 0.75 (0.51,1.1) | 0.136 |
| **DM** | 0.81 (0.69,0.96) | 0.015 | 0.85 (0.74,0.98) | 0.023 |
| **Liver disease** | 1.41 (1.19,1.66) | < 0.001 | 1.4 (1.22,1.62) | < 0.001 |
| **Renal disease** | 1.36 (1.15,1.62) | < 0.001 | 1.48 (1.28,1.71) | < 0.001 |
| **MV** | 2.72 (2.3,3.22) | < 0.001 | 2.12 (1.85,2.43) | < 0.001 |
| **APSⅢ** | 1.02 (1.02,1.02) | < 0.001 | 1.02 (1.01,1.02) | < 0.001 |
| **OASIS** | 1.03 (1.02,1.04) | < 0.001 | 1.03 (1.03,1.04) | < 0.001 |
| **TyG** | 1.17 (1.08,1.26) | < 0.001 | 1.11 (1.03,1.18) | 0.003 |

**Abbreviations:** BMI, body mass index; SBP, systolic blood pressure; DBP, diastolic blood pressure; Resp, respiratory; Spo_2,_ pulse oximetry derived oxygen saturation; Hb, hemoglobin; WBC, white blood cell; NE, neutrophil; PT, prothrombin time; INR, international normalized ratio; APTT, activated partial thromboplastin time; MI, myocardial infarction; CVD, cerebrovascular disease; PVD, peripheral vascular disease; COPD, chronic obstructive pulmonary disease; PUD, peptic ulcer disease; DM, diabetes mellitus; MV, mechanical ventilation; APSIII, acute physiology score III; OASIS, oxford acute severity of illness score; TyG, Triglyceride Glucose Index.
