# Supplementary material for: Triglyceride glucose index and mortality in tracheally intubated patients: a MIMIC-IV retrospective cohort study
Source: PLoS One. 2025 May 21;20(5):e0324162. doi: 10.1371/journal.pone.0324162 (PMC12094725; doi:10.1371/journal.pone.0324162)
Supplement: S2 Table — After excluding diabetic patients (n = 1816), the relationship between the TyG index and ICU mortality as well as in-hospital mortality. (DOCX) [file pone.0324162.s002.docx]

**TABLE S2. Relationships between the TyG index, ICU mortality, and in-hospital mortality across different models.**

| **Variable** | **Crude model** | | **Model Ⅰ** | | **Model Ⅱ** | | **Model Ⅲ** | |
| --- | --- | --- | --- | --- | --- | --- | --- | --- |
|  | **HR (95% CI)** | ***P-*value** | **HR (95% CI)** | ***P-*value** | **HR (95% CI)** | ***P-*value** | **HR (95% CI)** | ***P-*value** |
| **ICU mortality** |  |  |  |  |  |  |  |  |
| TyG as continuous | 1.28 (1.17~1.4) | <0.001 | 1.37 (1.26~1.5) | <0.001 | 1.27 (1.15~1.4) | <0.001 | 1.26 (1.14~1.39) | <0.001 |
| Quartiles |  |  |  |  |  |  |  |  |
| Q1 (TyG**<**8.76) | **Ref** |  | **Ref** |  | **Ref** |  | **Ref** |  |
| Q2 (8.76 ≤ TyG <9.25) | 1.25 (0.96~1.64) | 0.102 | 1.3 (0.99~1.7) | 0.058 | 1.32 (1~1.74) | 0.052 | 1.24 (0.94~1.64) | 0.133 |
| Q3 (9.25 ≤ TyG <9.92) | 1.17 (0.88~1.55) | 0.272 | 1.39 (1.05~1.84) | 0.023 | 1.28 (0.95~1.71) | 0.1 | 1.19 (0.88~1.61) | 0.248 |
| Q4 (TyG ≥9.92) | 1.99 (1.55~2.55) | <0.001 | 2.45 (1.9~3.15) | <0.001 | 1.96 (1.5~2.56) | <0.001 | 1.94 (1.48~2.54) | <0.001 |
| *P* for trend |  | <0.001 |  | <0.001 |  | <0.001 |  | <0.001 |
| **In-hospital mortality** |  |  |  |  |  |  |  |  |
| TyG as continuous | 1.15 (1.06~1.25) | <0.001 | 1.25 (1.15~1.35) | <0.001 | 1.16 (1.06~1.26) | 0.001 | 1.14 (1.04~1.24) | 0.003 |
| Quartiles |  |  |  |  |  |  |  |  |
| Q1 (TyG**<**8.76) | **Ref** |  | **Ref** |  | **Ref** |  | **Ref** |  |
| Q2 (8.76 ≤ TyG <9.25) | 1.24 (1~1.55) | 0.055 | 1.3 (1.04~1.62) | 0.019 | 1.22 (0.97~1.53) | 0.085 | 1.17 (0.93~1.47) | 0.186 |
| Q3 (9.25 ≤ TyG <9.92) | 1 (0.79~1.27) | 0.989 | 1.18 (0.93~1.49) | 0.178 | 1.16 (0.9~1.48) | 0.248 | 1.13 (0.88~1.46) | 0.328 |
| Q4 (TyG ≥9.92) | 1.6 (1.29~1.98) | <0.001 | 1.98 (1.6~2.46) | <0.001 | 1.61 (1.28~2.03) | <0.001 | 1.54 (1.22~1.94) | <0.001 |
| *P* for trend |  | <0.001 |  | <0.001 |  | <0.001 |  | <0.001 |

**Notes:** Crude model was not adjusted.

Model 1 was adjusted for age + sex.

Model 2 was adjusted for model 1 + BMI + heart rate + SBP + DBP + Resp + Spo_2_ + Hb + WBC + NE + PT + INR + APTT + MI + CVD + liver disease + renal disease + MV + APSⅢ + OASIS.

Model 3 was adjusted for model 2 + race + PVD + COPD + PUD.

**Abbreviations:** BMI, body mass index; SBP, systolic blood pressure; DBP, diastolic blood pressure; Resp, respiratory; Spo_2,_ pulse oximetry derived oxygen saturation; Hb, hemoglobin; WBC, white blood cell; NE, neutrophil; PT, prothrombin time; INR, international normalized ratio; APTT, activated partial thromboplastin time; MI, myocardial infarction; CVD, cerebrovascular disease; MV, mechanical ventilation; APSIII, acute physiology score III; OASIS, oxford acute severity of illness score; PVD, peripheral vascular disease; COPD, chronic obstructive pulmonary disease; PUD, peptic ulcer disease.
