# Supplementary material for: Triglyceride glucose index and mortality in tracheally intubated patients: a MIMIC-IV retrospective cohort study
Source: PLoS One. 2025 May 21;20(5):e0324162. doi: 10.1371/journal.pone.0324162 (PMC12094725; doi:10.1371/journal.pone.0324162)
Supplement: S3 Table — After including patients with hypertriglyceridemia (triglycerides ≥200 mg/dL) and those on lipid-lowering medications, the relationship between the TyG index and ICU mortality as well as in-hospital mortality. (DOCX) [file pone.0324162.s003.docx]

**TABLE S3. Relationships between the TyG index, ICU mortality, and in-hospital mortality across different models.**

| **Variable** | **Crude model** | | **Model Ⅰ** | | **Model Ⅱ** | | **Model Ⅲ** | |
| --- | --- | --- | --- | --- | --- | --- | --- | --- |
|  | **HR (95% CI)** | ***P-*value** | **HR (95% CI)** | ***P-*value** | **HR (95% CI)** | ***P-*value** | **HR (95% CI)** | ***P-*value** |
| **ICU mortality** |  |  |  |  |  |  |  |  |
| TyG as continuous | 1.17 (1.08~1.26) | <0.001 | 1.24 (1.15~1.34) | <0.001 | 1.17 (1.07~1.27) | 0.001 | 1.11 (1.01~1.21) | 0.03 |
| Quartiles |  |  |  |  |  |  |  |  |
| Q1 (TyG**<**8.83) | **Ref** |  | **Ref** |  | **Ref** |  | **Ref** |  |
| Q2 (8.83 ≤ TyG <9.37) | 1.21 (0.97~1.51) | 0.092 | 1.24 (0.99~1.55) | 0.058 | 1.34 (1.06~1.67) | 0.012 | 1.19 (0.94~1.49) | 0.148 |
| Q3 (9.37 ≤ TyG <10.03) | 1.19 (0.96~1.49) | 0.115 | 1.32 (1.05~1.65) | 0.015 | 1.32 (1.05~1.67) | 0.02 | 1.19 (0.94~1.51) | 0.149 |
| Q4 (TyG ≥10.03) | 1.53 (1.24~1.89) | <0.001 | 1.74 (1.4~2.15) | <0.001 | 1.48 (1.18~1.87) | 0.001 | 1.3 (1.02~1.66) | 0.035 |
| *P* for trend |  | <0.001 |  | <0.001 |  | 0.002 |  | 0.047 |
| **In-hospital mortality** |  |  |  |  |  |  |  |  |
| TyG as continuous | 1.11 (1.03~1.18) | 0.003 | 1.18 (1.1~1.26) | <0.001 | 1.13 (1.05~1.21) | 0.001 | 1.05 (0.97~1.14) | 0.192 |
| Quartiles |  |  |  |  |  |  |  |  |
| Q1 (TyG**<**8.83) | **Ref** |  | **Ref** |  | **Ref** |  | **Ref** |  |
| Q2 (8.83 ≤ TyG <9.37) | 1.18 (0.98~1.41) | 0.088 | 1.22 (1.01~1.47) | 0.035 | 1.24 (1.02~1.49) | 0.028 | 1.14 (0.94~1.38) | 0.177 |
| Q3 (9.37 ≤ TyG <10.03) | 1.05 (0.87~1.27) | 0.623 | 1.18 (0.97~1.42) | 0.096 | 1.2 (0.98~1.46) | 0.078 | 1.1 (0.9~1.35) | 0.34 |
| Q4 (TyG ≥10.03) | 1.38 (1.15~1.65) | <0.001 | 1.6 (1.33~1.92) | <0.001 | 1.41 (1.16~1.72) | 0.001 | 1.19 (0.97~1.46) | 0.104 |
| *P* for trend |  | 0.003 |  | <0.001 |  | 0.002 |  | 0.158 |

**Notes:** Crude model was not adjusted.

Model 1 was adjusted for age + sex.

Model 2 was adjusted for model 1 + BMI + heart rate + SBP + DBP + Resp + Spo_2_ + Hb + WBC + NE + PT + INR + APTT + MI + CVD + DM + liver disease + renal disease + MV + APSⅢ + OASIS.

Model 3 was adjusted for model 2 + race + PVD + COPD + PUD + HTG + LLD.

**Abbreviations:** BMI, body mass index; SBP, systolic blood pressure; DBP, diastolic blood pressure; Resp, respiratory; Spo_2,_ pulse oximetry derived oxygen saturation; Hb, hemoglobin; WBC, white blood cell; NE, neutrophil; PT, prothrombin time; INR, international normalized ratio; APTT, activated partial thromboplastin time; MI, myocardial infarction; CVD, cerebrovascular disease; DM, diabetes mellitus; MV, mechanical ventilation; APSIII, acute physiology score III; OASIS, oxford acute severity of illness score; PVD, peripheral vascular disease; COPD, chronic obstructive pulmonary disease; PUD, peptic ulcer disease; HTG, Hypertriglyceridemia; LLD, Lipid-lowering drugs.
