# Supplementary figures and images for: Triglyceride glucose index and mortality in tracheally intubated patients: a MIMIC-IV retrospective cohort study
Source: PLoS One. 2025 May 21;20(5):e0324162. doi: 10.1371/journal.pone.0324162 (PMC12094725; doi:10.1371/journal.pone.0324162)

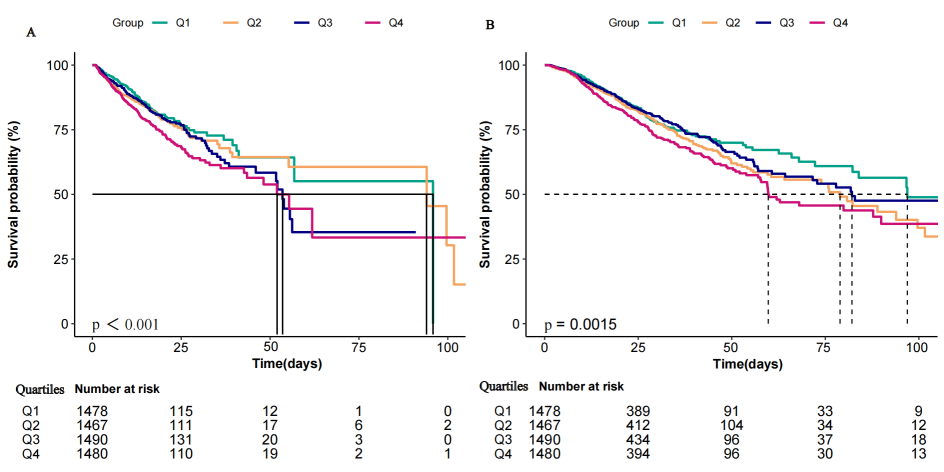

Supplement: S1 Fig — Kaplan-Meier survival analysis depicting the association between TyG and ICU mortality among patients who underwent tracheal intubation. The survival curves are stratified by TyG status, highlighting the differences in survival probabilities. B Kaplan-Meier survival analysis of TyG and in-hospital mortality in patients with tracheal intubation. This Kaplan-Meier survival plot examines the relationship between TyG and in-hospital mortality in patients with tracheal intubation. Indicating the impact of TyG and in-hospital mortality rates. (TIF) [file pone.0324162.s004.tif]
